# Direct Endoplasmic Reticulum Targeting by the Selective Alkylphospholipid Analog and Antitumor Ether Lipid Edelfosine as a Therapeutic Approach in Pancreatic Cancer

**DOI:** 10.3390/cancers13164173

**Published:** 2021-08-19

**Authors:** Faustino Mollinedo, Consuelo Gajate

**Affiliations:** Centro de Investigaciones Biológicas Margarita Salas, Consejo Superior de Investigaciones Científicas (CSIC), Laboratory of Cell Death and Cancer Therapy, Department of Molecular Biomedicine, C/Ramiro de Maeztu 9, E-28040 Madrid, Spain; consuelogajate@gmail.com

**Keywords:** endoplasmic reticulum stress, endoplasmic reticulum targeting, pancreatic cancer, cancer therapy, alkylphospholipid analog, edelfosine

## Abstract

**Simple Summary:**

Pancreatic ductal adenocarcinoma (PDAC), comprising 90–95% of all pancreatic cancers, is one of the deadliest human cancers, with a gloomy prognosis and ~6-month median survival in metastatic tumors. Even patients with resectable tumors show a poor survival rate after surgery. Thus, PDAC represents an unmet therapeutic challenge. The aim of this review was to put into context the conundrum of pancreatic cancer treatment and the advent of a novel therapeutic approach, examining: (a) anatomical factors, demographics, statistics, and therapeutic approaches, affecting tumor detection, treatment, and prognosis; (b) the importance of the endoplasmic reticulum as a major target for pancreatic cancer due to its high abundance and activity in pancreatic cells; (c) the identification of the alkylphospholipid analog edelfosine as a novel drug against pancreatic cancer, showing two outstanding features—selective uptake by tumor tissue, and direct accumulation in the endoplasmic reticulum, leading to persistent endoplasmic reticulum stress and subsequent apoptosis.

**Abstract:**

Pancreatic ductal adenocarcinoma (PDAC), the most common malignancy of the pancreas, shows a dismal and grim overall prognosis and survival rate, which have remained virtually unchanged for over half a century. PDAC is the most lethal of all cancers, with the highest mortality-to-incidence ratio. PDAC responds poorly to current therapies and remains an incurable malignancy. Therefore, novel therapeutic targets and drugs are urgently needed for pancreatic cancer treatment. Selective induction of apoptosis in cancer cells is an appealing approach in cancer therapy. Apoptotic cell death is highly regulated by different signaling routes that involve a variety of subcellular organelles. Endoplasmic reticulum (ER) stress acts as a double-edged sword at the interface of cell survival and death. Pancreatic cells exhibit high hormone and enzyme secretory functions, and thereby show a highly developed ER. Thus, pancreatic cancer cells display a prominent ER. Solid tumors have to cope with adverse situations in which hypoxia, lack of certain nutrients, and the action of certain antitumor agents lead to a complex interplay and crosstalk between ER stress and autophagy—the latter acting as an adaptive survival response. ER stress also mediates cell death induced by a number of anticancer drugs and experimental conditions, highlighting the pivotal role of ER stress in modulating cell fate. The alkylphospholipid analog prototype edelfosine is selectively taken up by tumor cells, accumulates in the ER of a number of human solid tumor cells—including pancreatic cancer cells—and promotes apoptosis through a persistent ER-stress-mediated mechanism both in vitro and in vivo. Here, we discuss and propose that direct ER targeting may be a promising approach in the therapy of pancreatic cancer, opening up a new avenue for the treatment of this currently incurable and deadly cancer. Furthermore, because autophagy acts as a cytoprotective response to ER stress, potentiation of the triggering of a persistent ER response by combination therapy, together with the use of autophagy blockers, could improve the current gloomy expectations for finding a cure for this type of cancer.

## 1. The Pancreas

**Figure 1:**
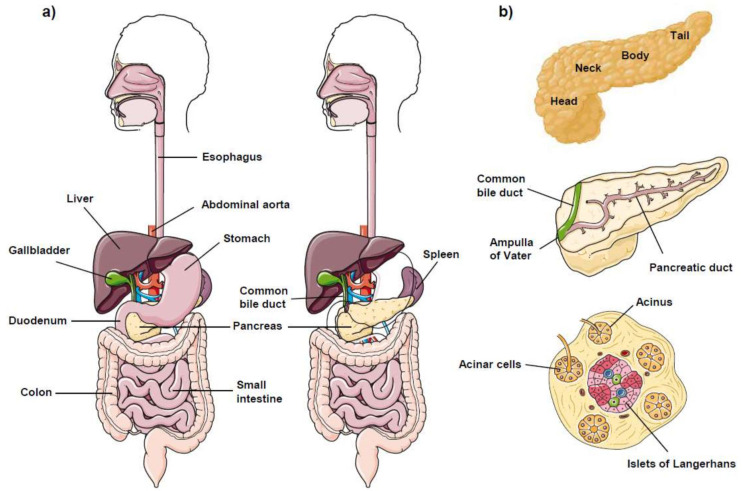
Pancreas anatomy. (**a**) Location and anatomical relationships between the pancreas and organs surrounding it in the abdomen. The pancreas is located behind the stomach, and the head of the pancreas is surrounded by the C-loop of the duodenum. (**b**) (Upper) The pancreas is divided into four major anatomical regions: head, neck, body, and tail. (Middle) The pancreatic duct extends from the tail to the head, and collects juices from all of the branches of the pancreatic stream. The pancreatic duct joins the common bile duct in the head of the pancreas to form the ampulla of Vater, which empties into the duodenum. (Lower) Most of the pancreas consists of exocrine tissue, producing pancreatic enzymes for digestion. The cells that synthesize and secrete digestive enzymes are clustered in grape-like bunches called acini (acinus, singular). These pancreatic acinar cells of the acinus synthesize, store, and secrete digestive enzymes that are drained into the pancreatic duct. The remaining tissue consists of endocrine cells called islets of Langerhans, which are clusters of pancreatic endocrine cells that produce and release hormones (such as insulin and glucagon) into the bloodstream regulating glucose levels. See text for details.

The pancreas is a very blood-rich, soft, and spongy glandular organ of both the digestive and endocrine systems that plays an essential role in turning the food we eat into fuel for our body’s cells. Etymologically, the origin of the word pancreas comes from the Greek πανκρέας (pankreas), meaning “all flesh” (παν, “all”; κρέας, “flesh”), apparently being named in this way for its meaty appearance. The pancreas serves as two types of gland—namely, a digestive exocrine gland (excreting a number of enzymes required to break down the proteins, lipids, and carbohydrates in food), and a hormone-generating endocrine gland (producing the blood sugar regulators insulin and glucagon). The pancreas should produce the above enzymes and hormones in the proper quantities and at the right time to digest and control the food we eat, and these functions of the pancreas are vital to the body’s survival. The pancreas is a flat and oblong-shaped gland, of about 15–25 cm (6–10 inches) long, extended horizontally across the back of the upper abdomen, and sits tucked behind the stomach [1] (Figure 1a). The pancreas can be divided in four major regions [1] (Figure 1b): head (the widest part of the pancreas, being located on the right side of the abdomen, at the juncture where the stomach meets the duodenum, and wrapped by the C shape of the duodenum); neck; body; and tail (the thin part of the pancreas on the left side of the abdomen, in close vicinity to the spleen).

## 2. Pancreatic Cancer

Approximately 65–75% of all pancreatic cancers occur within the head and neck of the pancreas, whereas ~15–25% occur in the body and tail; the remaining lesions diffusely involve the whole gland. Nevertheless, people with tumors in the body or tail of the pancreas have lower survival rates than those with cancer in the head or neck of the pancreas, independently of the presentation stage and extent of the disease [2,3].

The pancreas is the largest exocrine gland in the body, and is a unique gland that has both exocrine and endocrine functions [4]. The pancreas consists of 85–95% exocrine tissue (acinar and duct tissue), which secretes digestive enzymes into the duodenum, and less than 5% endocrine tissue (islets of Langerhans), which secretes hormones into the bloodstream [4,5] (Figure 1b). The exocrine pancreas comprises acinar, ductal, and centroacinar cells [6], which form the exocrine glands and ducts, producing enzymes that are secreted into the duodenum (Figure 1a,b). Ductal cells form an intricate network of small tubes (ducts), by which the acinar-cell-secreted enzymes (e.g., proteases, amylases, lipases) flow into the main pancreatic duct. The latter then joins the common bile duct (carrying bile from the liver and the gallbladder through the pancreas) to form the ampulla of Vater, through which the bile and pancreatic juices enter the duodenum to break down proteins, carbohydrates, and fats (Figure 1a,b). Most tumors affecting the exocrine gland are called adenocarcinomas. The vast majority of pancreatic cancers (~90–95%) initiate in the ducts of the pancreas, leading to the name of pancreatic ductal adenocarcinoma (PDAC) for the most common malignancy of the pancreas. Pancreatic acinar cells can undergo transdifferentiation to ductal-like cells (acinar-to-ductal metaplasia) during pancreatitis, and this might precede progression towards PDAC [7,8].

The endocrine pancreas consists of small islands containing endocrine cells (islets of Langerhans), which produce and release hormones (e.g., insulin and glucagon) into the bloodstream [6], thus controlling blood glucose levels (Figure 1b). A small percentage (1–2%) of all pancreatic cancers correspond to pancreatic neuroendocrine tumors (PanNETs or PNETs)—a diverse group of rare neoplasms previously referred to as islet cell tumors or pancreatic endocrine tumors, which have a slow and indolent growth [9]. PanNETs affect the secretion of pancreatic hormones and, therefore, they are commonly named after each specific hormone (gastrinoma, insulinoma, somatostatinoma, VIPoma, and glucagonoma, affecting cells producing gastrin, insulin, somatostatin, VIP, and glucagon, respectively) [10,11,12]. PanNET patients have a better prognosis than PDAC patients, with an overall median survival from diagnosis of 4.1 years for patients with PanNETs, whereas the median survival is less than 1 year for patients with PDAC [13].

The pancreas is located deep in the abdomen, with the head of the pancreas on the right side of the abdomen nestled in the C-loop of the duodenum, and the body and tail of the pancreas extending to the left side of the body behind the stomach. Due to this location, most pancreatic tumors are difficult to detect manually by pressing on the abdomen. Patients rarely display symptoms until an advanced stage of the disease, when the tumor interferes with the function of the pancreas or other nearby organs, such as the stomach, duodenum, liver, or gallbladder. Thus, by the time pancreatic tumors are detected, they are usually fairly large and metastatic—too late for surgical removal. 

**Figure 2:**
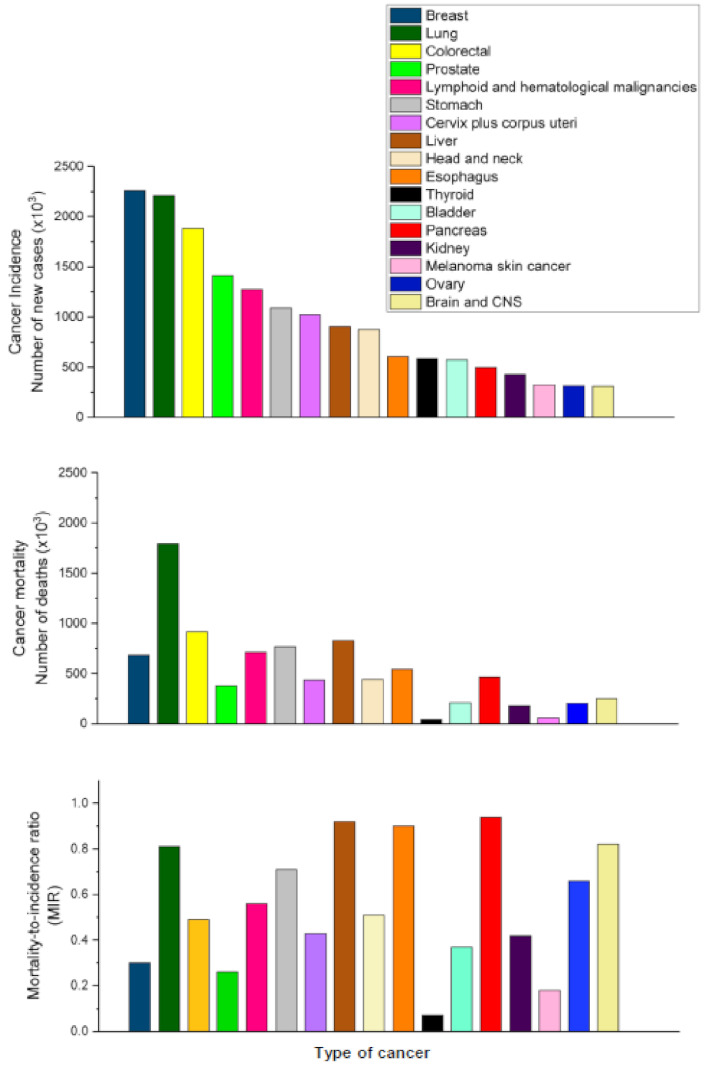
Cancer incidence, cancer mortality, and mortality-to-incidence ratios for the 17 most frequent cancers in 2020. The mortality-to-incidence ratio (MIR) is calculated by dividing the mortality count by the incidence count in a given year. Cancer MIR values were determined globally from incidence and mortality data obtained from the GLOBOCAN 2020 database. Colorectal cancers: colon; rectum. Hematological cancers: non-Hodgkin lymphoma; leukemia; multiple myeloma; Hodgkin lymphoma. Head and neck cancers: lip, oral cavity; larynx; nasopharynx; oropharynx; hypopharynx. CNS: central nervous system.

PDACs are indolent, difficult to treat tumors with one of the direst prognoses of any type of cancer (Figure 2), showing a rapid progress from diagnosis to death. Complete surgical resection remains the only potential curative treatment, but only 10–20% of pancreatic tumors are resectable at the time of presentation [14]. Unfortunately, the 5-year survival rate for patients with resectable PDAC remains rather low (15–20%) after surgical resection, likely as a result of metastatic disease or local recurrence [14]. 

PDAC shows an overall 5-year survival rate of 5–9%, depending on the specific stage of the disease when it is diagnosed [15,16,17,18,19]. From 2014–2018, the 5-year survival for PDAC barely increased, from 6% to 9% [19], which indicates that there is an urgent need to improve the survival rate.

To date, pancreatic cancer shows a dismal prognosis, with a mortality-to-incidence ratio of 0.94 (Figure 2)—this type of cancer accounting for almost as many deaths as cases (Figure 2). The incidence and mortality rates for PDAC are nearly equivalent (Figure 2), with the highest incidence rates in Europe, North America, and Australia/New Zealand—all of them belonging to the highest Human Development Index (HDI) countries [20,21,22]. Pancreatic cancer incidence is 4–5-fold higher in countries with elevated HDI [20,21,22,23]. There is a positive and significant correlation between incidence and mortality of pancreatic cancer and HDI, suggesting an association with socioeconomic development [24,25,26]. There is also a positive correlation between HDI and obesity—a major factor contributing to diabetes mellitus type 2 [27]. Obesity and diabetes are recognized risk factors for the development of pancreatic cancer [28,29], and the spread of obesity and diabetes worldwide could forewarn an increase in the incidence of pancreatic cancer [29].

Currently, pancreatic cancer is the seventh leading cause of global cancer deaths, and is the most lethal of the common malignancies [22,30]. When colon and rectal cancers are grouped together as colorectal cancer, and all patients with distinct hematological cancers are pooled together, pancreatic cancer ranks as the eighth leading cause of cancer death (Figure 2), with an estimated 466,000 deaths in 2020 worldwide [30].

Pancreatic cancer is a leading determinant of global cancer mortality, and it has been continuously rising in incidence in recent years. Pancreatic cancer is projected to become the third leading cause of cancer-related death in the European Union—after lung and colorectal cancers—by 2025 [31], and the second leading cause of cancer death in the United States by 2030 [32].

## 3. Differences in Mutated Genes between PDAC and PanNET

Although both tumor types are still incurable, the better prognosis of PanNET as compared to that of PDAC could be related to differences between their respective genetic profiles. More than 90% of PDAC cases at all grades carry mutated *KRAS* alleles [33], and none of the most frequently mutated genes in PDAC (*KRAS*, *CDKN2A*, *TP53, SMAD4*) are currently druggable [34]. Conversely, *KRAS* mutation is usually absent in PanNETs, which show ~60% fewer genes mutated per tumor than PADCs. The genes most commonly mutated in PDACs are barely faulty in PanNETs, and vice versa [35]. The most commonly mutated genes in PanNETs include *MEN1*, *DAXX*, *ATRX*, and *mTOR* [35,36].

## 4. Pancreatic Cancer Stages

Four different stages of pancreatic cancer have been defined, so as to describe the extent of the cancer’s spread and classify patients for treatment and clinical trials, namely:

Stage 0: No spread;

Stage I: Local growth. Tumor size is <2 cm (stage IA) or between 2–4 cm (stage IB);

Stage II: Local spread. Tumor is >4 cm, but limited to the pancreas; locally spread or spread to nearby lymph nodes. No spread to distant sites;

Stage III: Wider spread, including nearby blood vessels or nerves. No metastasis to distant sites;

Stage IV: Spread to distant organs.

Pancreatic cancer usually metastasizes to one or more organs and tissues located near the pancreas, such as the liver (leading to liver failure), as well as to various anatomical locations in the abdomen. PDAC patients are usually diagnosed at stage III or IV and, therefore, it has a very poor prognosis, with a usual 5-year survival of less than 8% [34]. Based on the above disease stages, pancreatic cancer can be considered to be either resectable (cancer has not spread and can be removable) or unresectable (cancer is locally advanced and has spread into major blood vessels, or it is already metastatic and has spread to other organs). Approximately 10–20% of pancreatic cancers could be considered resectable at diagnosis [37,38,39]. Currently, surgical resection remains the only curative treatment for pancreatic cancer, or option to achieve long-term survival. However, disappointingly, only modest improvements in survival have been accomplished [37,38,40]. Surgical resection improves survival, especially if accompanied with neoadjuvant therapy, but no cure is attained. Unfortunately, most of the patients with localized pancreatic cancer who undergo surgery will develop metastatic disease, regardless of whether or not they also undergo neoadjuvant therapy [41]. This suggests that surgery is not sufficient for a cure, and micrometastases, although clinically undetectable, are apparently present at the time of diagnosis [41]. Because it is difficult to diagnose pancreatic cancer at early stages, 80–90% of patients have unresectable tumors due to the advanced stage at diagnosis [19]. Patients undergoing resection usually develop recurrent tumors (69–75% of patients relapse within 2 years, and 80–90% relapse within 5 years) [42]. Lee et al. [39] found that the median survival was significantly longer with neoadjuvant therapy plus surgery (24 months), compared to surgery without neoadjuvant therapy (16 months) or palliative care (7 months). Christians et al. [43] found a median survival of 44.9 months in patients who completed neoadjuvant therapy plus surgery, compared with 8.1 months for the patients who were not resected. Cucchetti et al. [44] estimated that the mean entire lifespan for pancreatic cancer was 1.4 years, with surgical patients reaching a higher post-diagnostic lifespan (3.5 years) than non-surgical older individuals (0.8 years). Although surgical tumor resection remains the best option to improve survival, the increase in life expectancy is highly dependent on the patient’s age and adjuvant treatments in resected patients [45].

On the other hand, the current chemotherapeutic regimen available is limited and often ineffective [46,47], especially for PDAC, which is mostly diagnosed at stage III or IV. Early detection may be the key to reducing mortality, and may be supported by patients’ screening and prevention regimens [19].

## 5. Current Therapy against Pancreatic Cancer Is Ineffective

**Figure 3:**
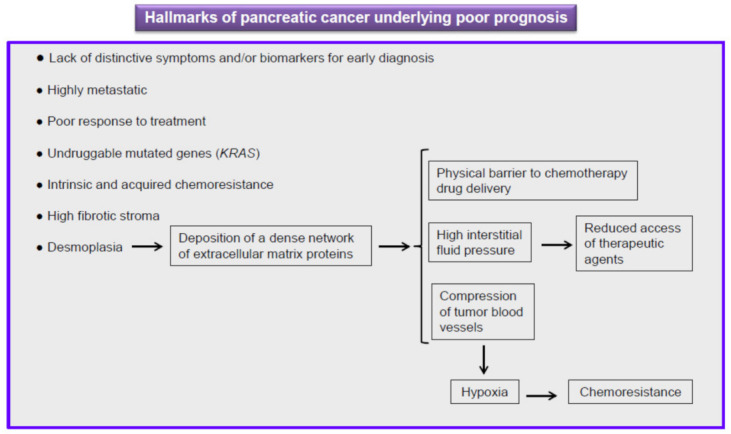
Major hallmarks in pancreatic cancer that affect chemotherapy and underlie poor prognosis.

Pancreatic cancer is an intractable malignancy and, as stated above, PDAC (accounting for over 90% of all cases of pancreatic) remains an incurable cancer, with its 5-year survival remaining dismally poor at ~5–9% [19,34,48], and the 1-year survival rate at less than 20% [49]. Outcomes have not improved substantially over the past four decades, and patients with locally advanced disease have a median survival of 6–10 months, while for those with metastatic disease it is only 3–6 months [19,48]. Several reasons lead to this grim prognosis [19,50,51] (Figure 3): (a) the lack of visible and distinctive symptoms and reliable biomarkers for early diagnosis; (b) aggressive metastatic spread; (c) the proportion of patients (around 50%) who show metastatic disease at diagnosis; (d) poor response to treatment, showing intrinsic and acquired chemoresistance; (e) tumor heterogeneity and plasticity facilitating resistance to cancer therapy; and (f) the progression of disease being accompanied by accumulating genetic alterations and aberrations in signaling pathways that likely make the tumor more aggressive and drug resistant.

Thus far, PDAC is considered to be the epitome of a treatment-resistant malignancy, likely driven by the thus-far “undruggable” KRAS oncoprotein [52,53]. At the time of diagnosis, ~10–20% of patients have resectable disease (stage I or II), ~35% have locally advanced pancreatic cancer (stage III), and ~50% have metastatic disease (stage IV) [54]. For a long time, the standard treatment for pancreatic cancer was restricted to the use of gemcitabine, with very limited benefit, and a modest survival improvement of a few months (3–6 months) [55,56,57].

A remarkable characteristic of pancreatic cancer is that the tumor microenvironment (tumor stroma) occupies up to 70–80% of the entire tumor mass, whereas tumor cells account for less than 20% of the total volume [58]. This leads to a strong degree of desmoplasia, which constitutes a fundamental pathological feature of pancreatic cancer (Figure 3). Desmoplasia is the result of a dramatic increase in the proliferation of cancer-associated fibroblasts/myofibroblasts and activated pancreatic stellate cells, resulting in increased deposition of extracellular matrix components. This high concentration of extracellular matrix molecules leads to a lack of tumor tissue elasticity and a concomitant increase in tumor interstitial fluid pressure which, in turn, leads to a decreased rate of perfusion of therapeutic agents and, consequently, reduced efficacy of chemotherapy [59] (Figure 3). Thus, this abundant, dense desmoplastic stroma is a major issue in pancreatic cancer chemotherapy, because antitumor drugs are considered to have difficulties penetrating this physical barrier, causing drug resistance [60]. The desmoplastic stroma of pancreatic cancer, mainly characterized by a dense extracellular matrix and abundant fibroblasts and inflammatory cells, is composed of several types of cells (e.g., fibroblasts, stellate cells, immune cells, and pericytes) and acellular components (e.g., fibrin, collagen, hyaluronic acid, fibronectin, growth factors, and cytokines), which lead to a tumor microenvironment characterized by low pH, hypoxia, and high tumor interstitial fluid pressure [61].

Treatment of metastatic pancreatic cancer relies on chemotherapeutic regimens, with FOLFIRINOX (folinic acid, 5-fluorouracil, irinotecan and oxaliplatin) and nanoparticle albumin-bound paclitaxel (nab-paclitaxel, Abraxane) plus gemcitabine as first-line therapies, and combinations of gemcitabine plus cisplatin and temsirolimus plus bevacizumab as second-line treatments, but the survival outcomes remain poor in all cases [53,62]. Thus, the development of novel and more effective treatments is urgently needed. Unfortunately, phase III clinical trials in PDAC show a very high failure rate (87%) [63], suggesting an insufficient robustness in the preclinical studies.

Since 1997, gemcitabine has been the standard first-line treatment for patients with a good Karnofsky performance status [64], but the response has been rather modest, with a median survival rate of a few months and a 1-year overall survival of ~18% [64]. The combination of gemcitabine and nab-paclitaxel (Abraxane) slightly improved the response in patients with advanced pancreatic cancer [65,66], leading to (a) a median overall survival of 8.5 months in the nab-paclitaxel–gemcitabine group vs. 6.7 months in the gemcitabine group, and (b) a survival rate of 35% in the nab-paclitaxel–gemcitabine group vs. 22% in the gemcitabine group at 1 year, and 9% vs. 4% at 2 years [65]. However, single-agent gemcitabine and its combinations have failed to meet expectations, prolonging life expectancy only moderately. In addition, the rather modest positive response to the Abraxane–gemcitabine therapy was accompanied by a considerable increase in the occurrence of adverse events, including grade ≥ 3 neutropenia (38% in the nab-paclitaxel–gemcitabine group vs. 27% in the gemcitabine group), febrile neutropenia (3% vs. 1%), thrombocytopenia (13% vs. 9%), fatigue (17% vs. 7%), and neuropathy (17% vs. 1%) [65]. Thus, the positive response of this combination, although rather modest, was accompanied by an increase in adverse effects [51,65,67]. Interestingly, recent data suggest that the nab-paclitaxel–gemcitabine treatment-induced neutropenia is associated with longer survival in metastatic pancreatic cancer patients [68].

Despite important achievements having been accomplished in the screening and treatment of other types of cancer, PDAC therapy has not significantly improved outcomes in recent decades. Gemcitabine and its combinations have failed to provide substantial survival benefits. The use of multidrug therapies (Abraxane and FOLFIRINOX) has only moderately improved patient outcomes. The efficacy of these dug combinations still remains low, and their use is linked to adverse effects [51]. Treatment with FOLFIRINOX achieved better efficacy parameters than gemcitabine, including overall survival (11.1 vs. 6.8 months) and 1-year survival rate (48.4% vs. 20.6%) [69], and led to a reduction in deterioration of quality of life when compared to gemcitabine-treated patients [70]. However, disappointingly, the toxicity profile of FOLFIRINOX treatment has tempered enthusiasm for its use, being associated mainly with neutropenia (grade 3 and 4), febrile neutropenia, and diarrhea [69,71]. FOLFIRINOX is considered to be a first-line option for advanced/metastatic pancreatic cancer, although its use is mainly constrained to patients under 75 years of age with a good performance status, due to its considerable toxicity [51]. Because of the high toxicity profile of most of the chemotherapeutic treatments against pancreatic cancer, patients’ Karnofsky performance status is one of the most important factors to take into account when starting drug treatment. Thus, learning how to manage the toxicity of these pharmacological treatments, or to reduce treatment toxicity, may improve their feasibility [51]. Several modified FOLFIRINOX treatments have been tested to decrease adverse events while providing survival benefits [72,73,74], but results have so far been rather modest.

A number of strategies to target specific oncogenes and related molecules, signaling pathways, growth factor receptors, angiogenesis, tumor–stroma interactions, and pancreatic stellate cells are being developed but, thus far, most of these targeted therapies have produced unsatisfactory results [51,75,76,77,78].

Tumor heterogeneity and plasticity are two common major obstacles in the treatment of pancreatic cancer, and disease progression is accompanied by accumulating morphological and genetic alterations, affecting cell proliferation, survival, and invasion [34,79,80,81]. As previously mentioned, activating mutations in *KRAS* constitutes a hallmark in the deadly and highly metastatic PDAC, occurring in 90–95% cases [82,83,84]. KRAS signals mainly through two signaling pathways—namely, the so-called RAF (rapidly accelerated fibrosarcoma)→MEK (MAPK/ERK kinase)→MAPK (mitogen-activated protein kinase)/ERK (extracellular signal-regulated kinase), and PI3K (phosphoinositide-3-kinase)→AKT→mTOR (mammalian target of rapamycin) signaling routes, which show extensive crosstalk [85]. Inhibition of the RAF→MEK→ERK signaling pathway in PDAC cells elicited autophagy as an adaptive and survival response. This might explain the lack of clinical benefit of MEK1/2 inhibitors (e.g., trametinib, pimasertib) in PDAC patients [86,87]. Xenografts in immunodeficient mice of human pancreatic cancer cell lines (MIA PaCa-2, BxPC-3), or tumor tissue from PDAC patients, were resistant to single-agent trametinib (MEK inhibitor) or chloroquine/hydroxychloroquine (autophagy inhibitor), but were sensitive to the combination of both inhibitors [88]. The combination of trametinib plus hydroxychloroquine yielded a partial disease response in a patient with metastatic PDAC refractory to standard-of-care therapies, including neoadjuvant modified FOLFIRINOX (mFOLFIRINOX), adjuvant gemcitabine/capecitabine, and palliative gemcitabine/Abraxane/cisplatin [88]. In this regard, the inhibition of autophagy (using chloroquine, or other genetic or pharmacologic inhibitors) potentiated the ability of ERK inhibitors to mediate antitumor activity in KRAS-driven PDAC [89]. Thus, inhibition of the ERK signaling pathway drives PDAC cells to be acutely dependent on autophagy, hence becoming highly sensitive to autophagy inhibitors. A number of different processes are also able to induce a high dependence of tumor cells on autophagy, thus providing new targets/drugs to be combined with autophagy inhibitors in pancreatic cancer treatment [85].

## 6. Aging and Pancreas

Aging is accompanied by several changes in pancreatic structure and function [90,91]. Pancreatic volume reaches its maximum (~78.85 cm^3^) in the third decade of life, and then pancreatic volume decreases with advancing age, reaching ~57.35 cm^3^ at the age of 70–80 years [90,92], while its hardness is increased [93], and the pancreatic ductal structure is dilated and shows a tendency to enlargement with advancing age [90,94]. An increased fibrosis around the acini, islets, and extracellular matrix is observed along with an apparent fatty infiltration [91,95,96]. At the ultrastructural level, several changes are observed with age, including mitochondrial swelling, vacuolization, and increased lipid droplets, lysosomes, and autophagosomes [91]. Downregulated islet cell function and decreased pancreatic exocrine function have been found in the elderly [90,91]. This inadequate pancreatic enzyme secretion, as a result of degenerative processes and gland damage, could lead to increased dyspeptic symptoms in the elderly. Thus, 5% of people older than 70 years and 10% older than 80 years show pancreatic exocrine insufficiency [97]. On the other hand, some age-related changes are consistent with a mild form of chronic pancreatitis called “senile pancreatitis”, which is often silent and very mild [90,97].

Considering, as stated above, that aging is frequently accompanied, to a variable degree, by a degeneration in pancreatic structure and function, and the increase in life expectancy, this could explain why the incidence rate of pancreatic cancer increases with age, and is estimated to increase in the coming years [19]. Pancreatic cancer rarely occurs before the age of 40 years, and is typically a disease of the elderly (≥70 years of age) [98], with the highest incidence peak occurring between 60 and 80 years of age [99]. Survival is negatively correlated with age at diagnosis, and patients aged <40 years diagnosed at an early stage have the best survival rate [100].

## 7. Endoplasmic Reticulum and Pancreas

Pancreatic acinar cells comprise about 80–90% of the pancreas, are highly specialized for the synthesis of digestive synthesis and secretion [101], and thereby have a highly developed, large, and abundant ER to meet the daily needs for protein synthesis. Their main function is to synthesize and secrete the hydrolytic enzymes that empty into the duodenum for the digestion of food, and they have the highest protein synthesis capacity in adult human tissues [102]. The pancreatic islets or islets of Langerhans make up just over 2% of the pancreatic tissue, and contain the endocrine (hormone-producing) cells of the pancreas with a well-developed ER, including glucagon-producing α-cells, insulin-producing β-cells (60–80% of the islet cell population), somatostatin-producing δ-cells, pancreatic-polypeptide-producing F (or PP)-cells, and ghrelin-producing ε-cells [103,104]. Thus, a hallmark of pancreatic cells is their highly developed ER, due to a high hormone and enzyme synthesis and secretion function [105].

## 8. ER Stress and Cancer Resistance

The ER is the main site of protein and lipid synthesis, membrane biogenesis, and cellular calcium storage, and any disturbance in ER homeostasis—including an imbalance between demand and capacity for protein synthesis and folding—leads to ER stress [106]. 

A number of circumstances favor the onset of an ER stress response in cancer cells, including a rapid cell proliferation leading to a protein build-up, inhibition of protein glycosylation, imbalance of ER calcium levels, as well as localized depletion of oxygen, nutrients, and glucose in the tumor microenvironment, which could be related to cancer chemoresistance [107]. This ER stress condition, characterized by the accumulation of misfolded proteins inside the ER lumen, triggers the unfolded protein response (UPR) [106,107], which is controlled by three ER transmembrane stress sensors—namely, inositol-requiring enzyme 1 (IRE1) [108], protein kinase RNA (PKR)-like ER kinase (PERK) [109], and activating transcription factor 6 (ATF6) [110]—with the main aim being to restore the ER’s physiological activity. This UPR signal transduction is fundamentally a pro-survival process, although sustained or protracted ER stress may result in cell death [106]. This reflects the critical importance of the timing of the process, and this is particularly evident in the case of an ER stress response, which is pro-survival in a short-term response, while it turns into a deadly response in a protracted or persistent response.

An ER stress response also plays a role in the PDAC-specific adaptive immune response, selecting quiescent, single disseminated cancer cells, which are resistant to immune elimination, thus enabling cancer PDAC cells to escape immunity and facilitate metastasis [111].

Sustained ER stress is induced in chronic pancreatitis [112], and pancreatic tumors tend to show high basal levels of ER stress [107]. Furthermore, autophagy seems to play an essential role in PDAC survival and progression, likely providing fuel to pancreatic cancer cells in their nutrient-deprived environment [113]. In this regard, ER stress could lead to an increase in autophagy to keep cells alive in nutrient-deprived media [114]. ER stress response can also induce dormancy in PDAC [115] which, if conveniently modulated, could provide a potential treatment for the emergence of distant metastasis and prolong patient survival [116].

## 9. ER Stress and Antitumor Drugs

A number of antitumor drugs have been reported to act through an ER stress response in different human cancers, including bortezomib (also known as Velcade or PS-341) [117,118], cisplatin [119], cannabinoids [120], and a wide number of plant-derived natural compounds, such as curcumin, resveratrol, green tea polyphenols, tocotrienols, and garcinia derivatives [121]. Bortezomib is a selective and potent inhibitor of the 26S proteasome, and proteasome inhibition can promote an accumulation of misfolded proteins in the cell, leading to ER stress [117]. Bortezomib promotes apoptosis triggered by classic ER stress inducers (such as tunicamycin—an inhibitor of protein glycosylation—and thapsigargin—an inhibitor of sarco/endoplasmic reticulum Ca^2+^-ATPases) via a c-Jun NH_2_-terminal kinase (JNK)-dependent mechanism [122]. The combined use of bortezomib plus cisplatin induced JNK activation and apoptosis in orthotopic pancreatic tumors, leading to a reduction in tumor burden [122]. Bortezomib sensitized pancreatic cancer cells to ER-stress-induced apoptosis, interacted with cisplatin to increase ER dilation, and strongly enhanced the anticancer activity of cisplatin [122].

**Figure 4:**
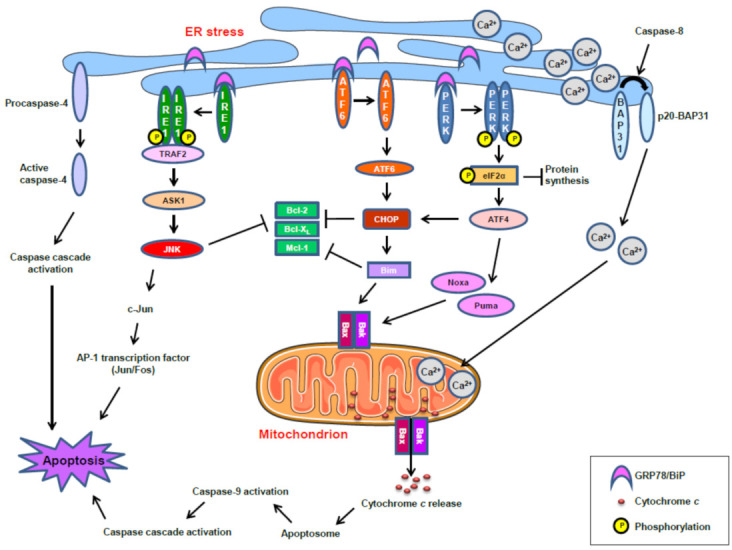
Persistent ER stress-induced apoptosis. Following a sustained ER stress, all three ER stress sensors (IRE1, ATF6, and PERK) activate a series of signaling events depicted in this scheme, involving transcriptional and post-translational modifications, which eventually lead to apoptotic cell death. A crosstalk between the ER and mitochondria is critical for cell demise. Most, if not all, of the signals converge in the mitochondria, before triggering the final cell death response. Additional processes involving BAP31-mediated Ca^2+^ release from the ER to the mitochondria, and the activation of caspase 4, also play major roles in the onset of the apoptotic response. See text for details.

However, in the long term, the development of bortezomib resistance seems to be generated through induction of the UPR pathway, associated with ER stress, as an adaptive cellular program to cope with protein misfolding stress in both hematological and solid cancers [123,124]. Thus, ER-stress-induced activation of the UPR represents a mechanism of protein quality control in the ER and cytoprotection [125,126]. However, unresolved and persistent ER stress leads to cell death by apoptosis [127,128]. Figure 4 shows schematically the major processes and signaling routes involved in the induction of apoptosis mediated by an ER stress response, based on previously reported studies [114,128,129,130,131,132,133,134,135,136]. In response to ER stress, the mammalian UPR is initiated by three ER-resident transmembrane protein sensors: namely, IRE1, ATF6, and PERK. The ER chaperone GRP78 (78-kDa glucose-regulated protein) (also known as BiP: binding immunoglobulin protein) is a master regulator of ER homeostasis, facilitating the folding and assembly of nascent polypeptides, preventing protein misfolding and aggregation, targeting misfolded proteins for proteasome degradation, and controlling the initiation of the various arms of the UPR, acting as a repressor of UPR stress sensors through direct binding to them [133,137,138] (Figure 4). IRE1 becomes active when monomers oligomerize into dimers or higher order structures, leading to transautophosphorylation and downstream signaling through the TRAF2→ASK1→JNK pathway, which could promote apoptosis, as shown in Figure 4. Under ER stress, PERK also oligomerizes into dimers, leading to transautophosphorylation and downstream signaling through the eIF2α→ATF4→CHOP pathway. The hyperactivation of PERK upregulates CHOP which, in turn, inhibits the expression of antiapoptotic Bcl-2 members, whereas it enhances the expression of proapoptotic Bcl-2 members, such as Bim [136,139] (Figure 4). Under ER stress, ATF6, after dissociating from GRP78, translocates to the Golgi, where it is cleaved by the proteases S1P and S2P, thereby liberating the N-terminal cytosolic fragment of ATF6 that causes upregulation of a number of UPR genes and other genes that could affect cell fate [128,136] (Figure 4). 

In addition, the integral protein of the ER B-cell receptor-associated protein 31 (BAP31) can be cleaved into the 20-kDa fragment p20-BAP31 following chronic ER stress [132], thus directing proapoptotic signals between the ER and mitochondria [140,141], and causing Ca^2+^ release from the ER, followed by Ca^2+^ entry into the mitochondria which, in turn, induces apoptosis [140,141]. On the other hand, caspase 4 has been shown to function as an ER-stress-specific caspase in humans [130,142].

As stated above, malignant cells have higher protein synthesis rates than normal cells [143] and, therefore, they are more prone to undergoing protein build-up and misfolding processes, leading to ER stress and, eventually, to apoptosis, when the ER function is persistently compromised [105]. Particularly, pancreatic epithelial cells exhibit a highly developed ER, because of their heavy engagement in hormone and digestive enzyme synthesis and secretion [105], and this fact makes pancreatic cells especially sensitive to ER-stress-induced apoptosis [144,145]. 

## 10. Alkylphospholipid Analogs as Selective Antitumor Drugs against Cancer Cells by Targeting Subcellular Structures

**Figure 5:**
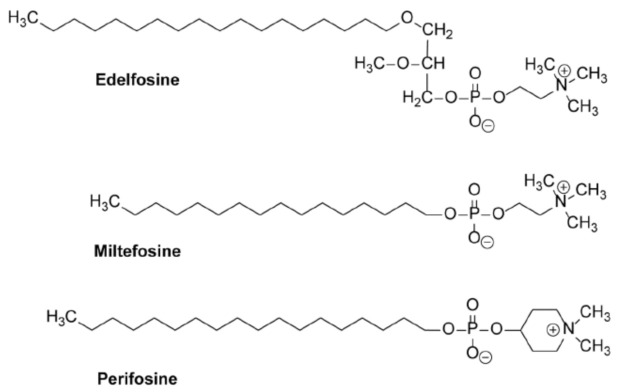
Chemical structures of some clinically relevant alkylphospholipid analogs (APLs).

Taking into account that the timing and intensity of ER stress are critical factors in promoting survival or an apoptotic response, the selective induction of a persistent ER stress response in malignant cells, while normal cells are spared, could constitute a hypothetical promising approach in fighting pancreatic cancer. In this context, some members of a family of synthetic chemical compounds, collectively known as alkylphospholipid analogs (APLs), draw particular attention. APLs—also known as alkyl-lysophospholipid analogs (ALPs), antitumor ether lipids (AELs), or antitumor lipids (ATLs) [146,147,148,149,150]—were initially synthesized as metabolically stable analogs of 2-lysophosphatidylcholine [146]. These drugs have been shown to elicit important pharmacological activities against several diseases, including leishmaniasis—particularly miltefosine (hexadecylphosphocholine) (Figure 5) [148,151,152,153,154]—and cancer—particularly perifosine (Figure 5) (octadecyl-[1,1-dimethyl-piperidino-4-yl]phosphate) in clinical trials for hematological [148,155,156,157,158,159,160] and solid tumors [148,161,162,163,164,165,166,167]. A number of APLs have shown interesting biomedical activities, and are promising drugs in the treatment of different diseases in the clinic [148]. Miltefosine, representing the minimal structural requirement for the antitumor activity of APLs, has become the first oral drug to treat visceral and cutaneous leishmaniasis [152,154,168], marketed under the trade name of Impavido [148]. Miltefosine has also been approved to be used in the clinic as a topical drug, marketed under the trade name of Miltex (6% miltefosine solution), for the palliative treatment of cutaneous metastases from breast cancer [148,169].

Edelfosine (1-*O*-octadecyl-2-*O*-methyl-*rac*-glycero-3-phosohocholine; formerly named ET-18-OCH_3_) (Figure 5) is considered to be the prototype molecule of APLs, and studies to elucidate its mechanism of action have been extrapolated to other APLs [150,170]. Edelfosine was first synthesized in 1969 by Günter Kny [171], a chemical diploma student in Otto Westphal’s group, following the previous studies of Bernd Arnold and Hans Ulrich Weltzien synthesizing 1-*O*-alkyl- and 2-*O*-methyl derivatives of glycerol [146]. The molecular structure of edelfosine is critical for its biological actions [146,147,172], and the presence of the ether bonds in C1 and C2 of the glycerol backbone—instead of the typical ester bonds in a phospholipid molecule—makes this ether lipid very metabolically stable in comparison with its natural counterparts [147]. As a matter of fact, more than 98% of edelfosine remained unmodified inside the tumor cells after 24 h of incubation [173,174], thus indicating that edelfosine is active per se, and does not act as a prodrug. 

Studies on edelfosine are paving the way for the synthesis of new APLs with promising therapeutic biomedical properties [149]. Edelfosine shows a higher antileishmanial activity than miltefosine [175,176]. Edelfosine also shows additional antiparasitic activities, including anti-*Schistosoma mansoni* [177,178] and anti-*Strongyloides venezuelensis* [179] activities. When it comes to cancer, and despite new generations of APLs having been synthesized, edelfosine shows the highest antitumor activity against a wide number of different hematological and solid tumors [132,135,146,170,180]. In addition, in vivo experiments with animal models show that edelfosine is very effective against a number of tumors, including multiple myeloma [181], chronic lymphocytic leukemia [180], mantle-cell lymphoma [182], Ewing’s sarcoma [135], and pancreatic cancer [132]. On the other hand, edelfosine lacks significant toxicity, not showing any significant cardiotoxicity, hepatotoxicity, or renal toxicity [183].

A number of key findings on the mechanism of action of APLs were unveiled in the late 1990s and early 2000s by Faustino Mollinedo and Consuelo Gajate’s research group, first in Valladolid (Spain) and then in Salamanca (Spain), showing that edelfosine was able to induce apoptosis selectively in cancer cells, whereas normal, non-transformed cells were spared [172,184]. The underlying basis for this selectivity was the preferential uptake of edelfosine by tumor cells when compared to normal, non-transformed cells [146,172,184,185]. Shortly after, we found that its antitumor action—especially in hematological cancer cells—was due to the reorganization of membrane raft domains [180,181,184,186,187,188,189,190,191,192,193], leading for the first time to the activation of the Fas/CD95 death receptor from within the cell, independently of its ligand FasL/CD95L, through its recruitment in membrane raft scaffolds [184,186]. Subsequent studies led to a further characterization of these edelfosine-induced co-clusters of Fas/CD95 and membrane rafts, which were enriched in proapoptotic downstream signaling molecules [170,184,186,188,194,195,196], while survival signaling molecules were displaced from lipid rafts [189,193,197,198,199,200]. These data were the first evidence for a selective proapoptotic drug, as well as for the involvement of membrane rafts as a major target in cancer chemotherapy and as major regulators of apoptosis [187,193].

Interestingly, while edelfosine accumulated in membrane lipid rafts in hematological cancer cells, leading to a rapid induction of apoptosis [180,181,184,188], this ether lipid mainly accumulated in the ER of several solid tumors [132,135,201], promoting a slower induction of apoptotic cell death. Taking together, edelfosine behaves as a promising antitumor drug that targets membrane domains (membrane lipid rafts) and subcellular structures (ER) rather than specific molecules, thus expanding the possibilities to achieve a stronger and deeper effect on a target cell by affecting several molecules and signaling processes simultaneously instead of individual proteins. 

Regarding pancreatic cancer, edelfosine showed a potent in vitro and in vivo antitumor activity against different human pancreatic cancer cell lines [132]. When compared with additional APLs of clinical interest, APLs ranked as follows: edelfosine > perifosine >> erucylphosphocholine ≥ miltefosine, regarding the ability to induce apoptosis in different human pancreatic cancer cell lines, including BxPC-3, Capan-2, CFPAC-1, and HuP-T4 [132].

## 11. Accumulation of the Ether Lipid Edelfosine in the ER of Pancreatic Cancer Cells

It is interesting to note that edelfosine behaves as a potent antitumor drug, promoting apoptosis in multiple myeloma (MM) [170,181,202] and pancreatic cancer [132] cells—two tumors showing a highly developed ER and a very high level of protein synthesis—through an excessive production of monoclonal immunoglobulins (also referred to as paraproteins) in MM cells [124,203] and of insulin and digestive enzymes in pancreatic cells. Thus, in the ER, both tumors seem to have an “Achilles’ heel” that could be exploited as a therapeutic target.

ER stress and its downstream cell death pathways can be engaged by a wide array of stimuli (e.g., thapsigargin, tunicamycin, brefeldin A, 2-deoxyglucose, chronic exposure to long-chain free fatty acids, cannabinoids, several plant-derived natural compounds) and anticancer drugs (e.g., bortezomib) in different cell types, including pancreatic cells [120,121,128,204,205,206]. However, this induction of the ER stress response is usually indirect, through the action on different processes that eventually lead to an accumulation of unfolded proteins.

**Figure 6:**
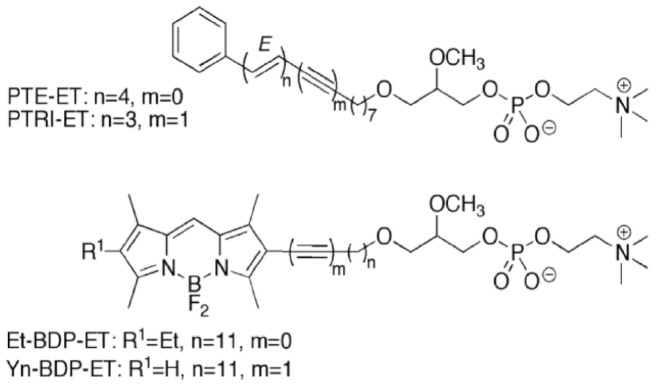
Chemical structures of fluorescent edelfosine analogs.

Unlike the above stimuli and anticancer drugs, edelfosine is selectively taken by tumor cells [184,185], and rapidly accumulates in the ER—especially in solid tumor cells—leading to persistent ER stress and, eventually, to apoptosis [132,135,201]. In order to visualize the subcellular localization of edelfosine, we used different newly synthesized fluorescent edelfosine analogs, including the first fluorescent edelfosine analog all-(*E*)-1-*O*-(15′-phenylpentadeca-8′,10′,12′,14′-tetraenyl)-2-*O*-methyl-*rac*-glycero-3-phosphocholine (PTE-ET) [184,207], its variant PTRI-ET, and the second generation of fluorescent edelfosine analogs that contained BODIPY (4,4-difluoro-4-bora-3a,4a-diaza-*s*-indacene; boron-dipyrromethene) [208,209], as a very potent fluorescent label incorporated into the alkyl chain of edelfosine, leading to the edelfosine analogs 1-*O*-[11′-(6”-ethyl-1”,3”,5”,7”-tetramethyl-4”,4”-difluoro-4”-bora-3a”,4a”-diaza-*s*-indacen-2”-yl)undecyl)]-2-*O*-methyl-*rac*-glycero-3-phosphocholine (Et-BDP-ET) and 1-*O*-[13′-(1”,3”,5”,7”-tetramethyl-4”,4”-difluoro-4”-bora-3a”,4a”-diaza-*s*-indacen-2”-yl)tridec-12′-ynyl]-2-*O*-methyl-*rac*-glycero-3-phosphocholine (Yn-BDP-ET) [210] (Figure 6).

**Figure 7:**
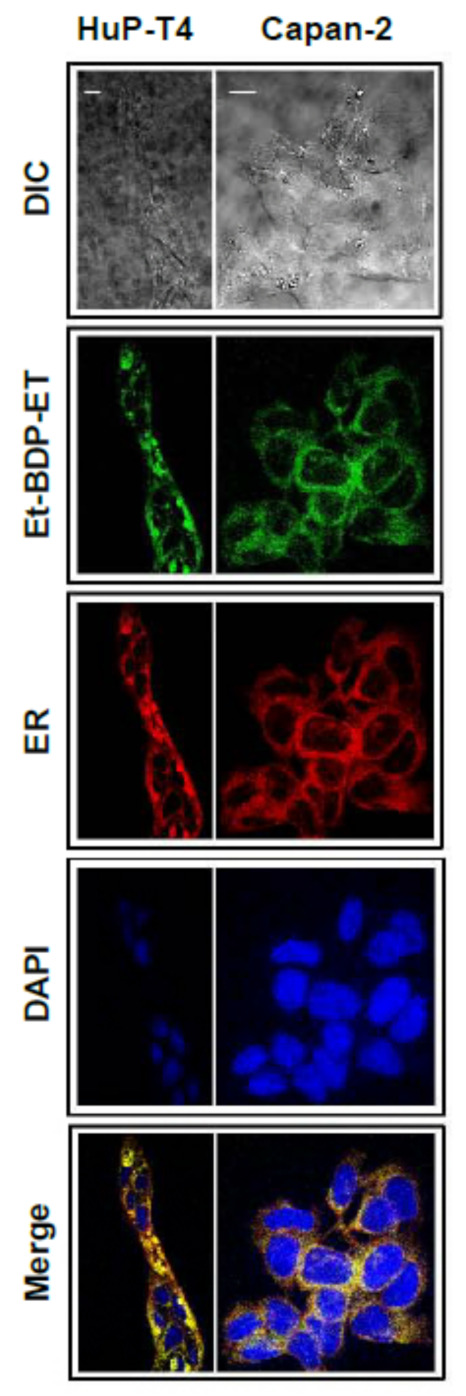
Edelfosine colocalizes with the ER in human pancreatic cancer cells. HuP-T4 and Capan-2 pancreatic cancer cells, transfected with an ER-targeted red fluorescence calreticulin plasmid to visualize the ER (red fluorescence), were incubated with the fluorescent edelfosine analog Et-BDP-ET (green fluorescence). Cell nuclei were also stained with the fluorophore 4′,6-diamidino-2-phenylindole (DAPI) (blue fluorescence). Areas of colocalization between the ER and Et-BDP-ET in the merge panels are yellow. Bar, 10 μm. DIC: differential interference contrast microscopy. See text for details. Image taken from [132].

Using the BODIPY fluorescent edelfosine analog Et-BDP-ET [210], we found that this BODIPY-conjugated fluorescent derivative accumulated in the ER of HuP-T4 and Capan-2 human pancreatic cancer cells [132] (Figure 7). This ER localization of the BODIPY fluorescent edelfosine analog was also found in Ewing’s sarcoma cells [135], and the fluorescent PTE-ET edelfosine analog mainly accumulated in the ER of human cervical carcinoma HeLa cells, non-small-cell lung cancer A549 cells, and glioblastoma cells [201], as assessed using a version of a red fluorescent protein (RFP) targeting the ER that colocalized with the ER marker calreticulin [211]. 

**Figure 8:**
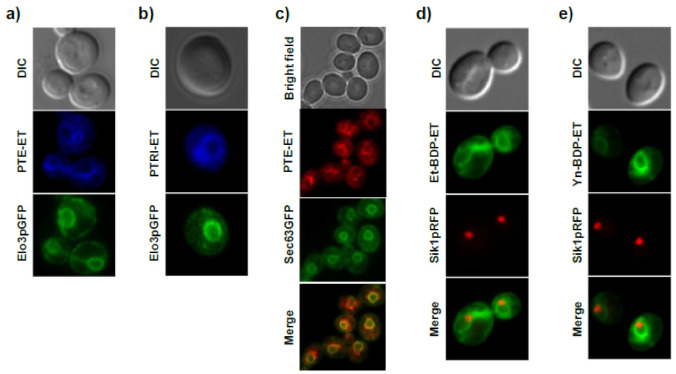
Localization of edelfosine in the ER of *Saccharomyces cerevisiae*. The fluorescent edelfosine analogs (**a**) PTE-ET and (**b**) PTRI-ET localize in the ER, as shown by their colocalization with the ER marker Elo3pGFP. (**c**) The fluorescent edelfosine analog PTE-ET (pseudocolored red) localizes in the ER, as shown by its colocalization with the ER marker Sec63GFP. Areas of colocalization between ER and PTE-ET in the merge panels are yellow. The corresponding bright field and differential interference contrast (DIC) images are also shown. (**d**,**e**) The fluorescent edelfosine analogs (**d**) Et-BDP-ET and (**e**) Yn-BDP-ET localize in the ER, as assessed by their visualization around the nucleolus marker Sik1pRFP, and close to the vacuole, as seen by DIC microscopy. See text for details. Images taken from [200].

In an attempt to unveil the molecular mechanism of the antitumor ether lipid edelfosine, we used the budding yeast *Saccharomyces cerevisiae*—which is sensitive to the cytotoxic action of edelfosine [197,199,200,212]—as an experimental eukaryotic model organism [213]. We found that different fluorescent edelfosine analogs (PTE-ET, PTRI-ET, Et-BDP-ET, and Yn-BDP-ET) (Figure 6) yielded a similar localization pattern in yeast (Figure 8) [200]. This pattern of fluorescent edelfosine consisted of a bag-like envelope surrounding the nucleus (identified by the nucleolus marker Sik1p tagged with RFP), which resembled the morphological traits of the ER (Figure 8) [200]. In fact, we found an excellent colocalization between fluorescent edelfosine and the ER, as assessed by using Elo3p-GFP or Sec63pGFP as ER markers (Figure 8) [200]. No colocalization was detected between fluorescent edelfosine and other subcellular organelles or compartments, such as the Golgi, spindle pole, endosomes, cytoskeleton, peroxisomes, and lipid droplets [200]. Quantification of the subcellular localization of fluorescent edelfosine in *S. cerevisiae* showed that most of the antitumor ether lipid accumulated in the ER, albeit an additional localization was found in the yeast [200]. Interestingly, edelfosine binds to lipid rafts in both human cells [180,181,184,188] and yeast [199,200]. As a matter of fact, edelfosine was mainly found in the ER after 3 h of incubation with *S. cerevisiae*, but a significant amount of the antitumor drug was also found in lipid rafts, as determined after sucrose gradient raft isolation [199,200], which could suggest the presence of lipid rafts in the ER [200]. On the other hand, the ER localization of edelfosine in yeast did not depend on endocytosis, as PTE-ET could still be detected in endocytosis-defective *end4pep4*Δ mutants [200]. Thus, it is tempting to envisage the existence of traffic between the plasma membrane and the ER through lipid rafts. In this regard, it is worth remembering that the ER is a large intracellular organelle extending from the nuclear envelope to the cell membrane.

Targeting of a subcellular structure offers the advantage of simultaneously affecting several proteins and signaling processes, as compared to the targeting of only one specific molecule or signaling process. In this way, global processes can be affected, and a stronger and longer lasting response can be achieved. Thus, targeting the ER could lead to a potent response, eventually leading to cell death. However, it might be reasonable to think that a concomitant higher cytotoxicity could also be a major and serious side effect, as the ER is present in both cancer and non-cancer cells. In the case of edelfosine, this latter problem is averted, because this antitumor ether lipid acts from within the cell [184,185], and it is taken up preferentially by cancer cells, while non-cancer cells are spared [146,147,170,184]. 

## 12. Induction of ER Stress-Mediated Apoptosis following Treatment with the Ether Lipid Edelfosine in Pancreatic Cancer Cells 

**Figure 9:**
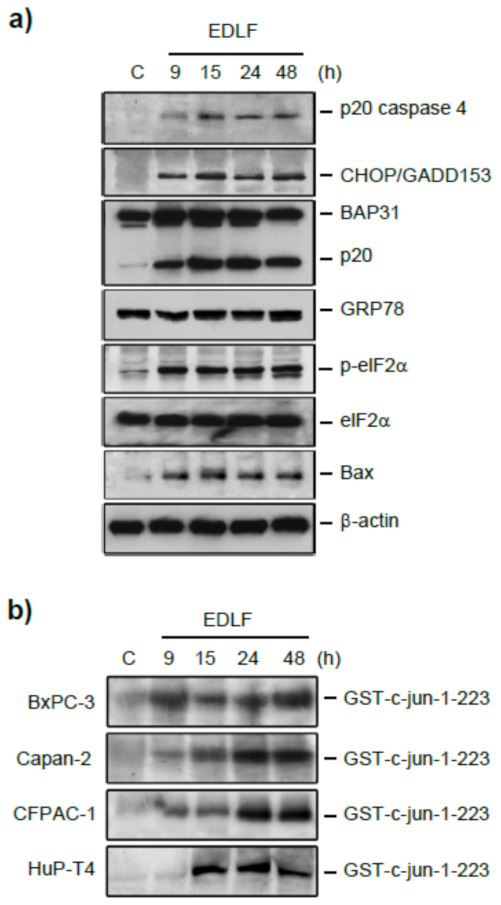
Edelfosine induces ER stress in human pancreatic cancer cells. (**a**) HuP-T4 cells, untreated (C) or treated with 20 μM edelfosine (EDLF) for the indicated times, were analyzed via Western blot using specific antibodies for the indicated proteins and ER stress markers; β-actin was used as a loading control. (**b**) The indicated pancreatic cancer cells, untreated (C) or treated with 20 μM edelfosine (EDLF) for the indicated times, were analyzed for JNK activation, as assessed by a solid-phase JNK assay using GST-c-Jun-1-123 as a substrate. See text for details. Images taken from [132].

As shown above, edelfosine accumulates in the ER of human pancreatic cancer cells, as assessed by using the fluorescent edelfosine analog Et-BDP-ET [132] (Figure 7). This leads to a potent induction of an ER stress response (Figure 9), characterized by an upregulation of the transcription factor CCAAT/enhancer-binding protein homologous protein (CHOP) (also known as growth-arrest and DNA-damage-inducible gene 153 (GADD153)), an increase in the phosphorylation level of eIF2α, and activation of caspase 4, Bax, and JNK [132] (Figure 9). In addition, the integral membrane protein of the ER (BAP31) is cleaved into the 20-kDa fragment p20-BAP31 [132], which directs proapoptotic signals between the ER and the mitochondria [140]. However, the ER chaperone GRP78, linked to a cytoprotective response [214,215], is not upregulated following edelfosine treatment [132]. Similar ER stress responses are also attained when edelfosine induces apoptosis in additional tumor cells [135,201,216]. The balance between GRP78 (cytoprotective) and CHOP/GADD153 (proapoptotic) may lead to either cell survival or cell death following an ER stress response [217]. Edelfosine upregulates CHOP/GADD153, without any change in the GRP78 protein level (Figure 9), thus tipping the GRP78/CHOP balance in favor of ER-stress-induced cell death. When ER stress is too severe or chronic, a series of ER-stress-induced apoptosis routes are activated, leading to JNK and caspase activation and CHOP upregulation, as well as Bax and Bak activation [217]. Edelfosine meets all of these requirements in its proapoptotic action in pancreatic cancer [132] (Figure 9a,b) and other tumors [135,201,216].

A remarkable feature of edelfosine is its ability to induce a dramatic activation of JNK in cancer cells [132,216,218]. Edelfosine promotes a very potent and persistent JNK activation that precedes the onset of apoptosis [218], as well as a high increase in the c-*jun* mRNA level that is associated with the activation of activator protein-1 (AP-1) transcription factor [218]. Edelfosine analogs that did not induce apoptosis failed to activate JNK, and there was a good correlation between edelfosine-induced apoptosis and the degree of JNK activation [218]. Specifically, edelfosine induces a dramatic activation of JNK, as assessed by a solid-phase JNK assay using a fusion protein between glutathione-S-transferase (GST) and c-Jun (amino acids 1–223) as a substrate for JNK-mediated phosphorylation, in a wide number of human pancreatic cancer cells, including BxPC-3, Capan-2, CFPAC-1, and HuP-T4 [132] (Figure 9b). Pretreatment with the JNK-specific inhibitor SP600125 [219] diminished both edelfosine-induced JNK activation and apoptosis [135,216,219]. Gene-transfer-mediated overexpression of apoptosis signal-regulating kinase 1 (ASK-1), which plays a crucial role in ER stress [220], increased edelfosine-induced JNK activation and apoptosis [216], supporting the involvement of ASK1/JNK signaling in the ER-stress-induced apoptosis triggered by edelfosine. As a novel mechanism favoring JNK activation, we found a new chaperoning role of heat shock protein 90 (Hsp90) on JNK-mediated apoptosis, following the recruitment of Hsp90 and JNK in lipid rafts, as assessed by lipid raft isolation followed by co-immunoprecipiation as well as by immunoelectron microscopy in hematological cancer cells [221]. 

Edelfosine treatment also inhibits phosphatidylcholine and protein synthesis in tumor cells [216], suggesting that this drug affects both phospholipid and protein homeostasis, and prevents the accumulation of newly synthesized proteins into the ER when the ER itself is compromised.

**Figure 10:**
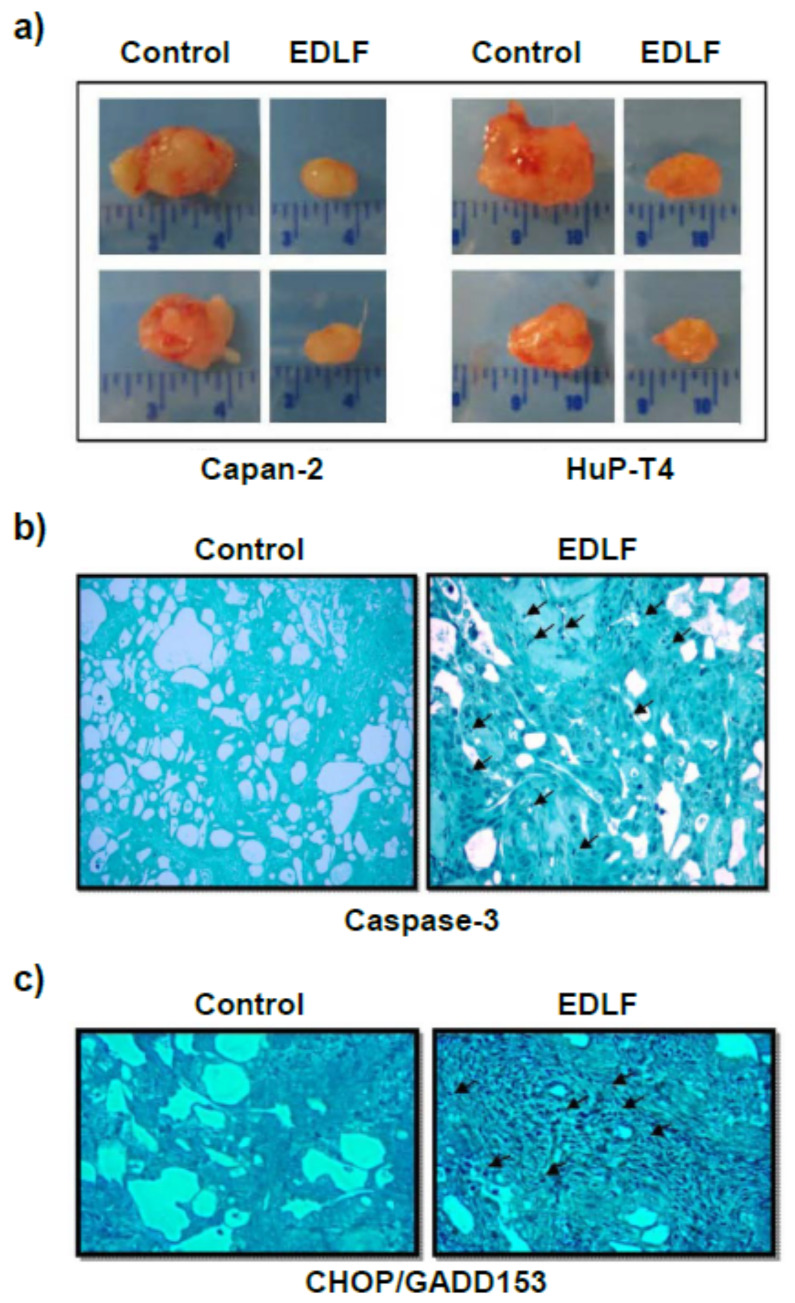
In vivo antitumor activity of edelfosine on human pancreatic cancer xenograft models. (**a**) CB17 SCID mice were inoculated subcutaneously with Capan-2 or HuP-T4 pancreatic cancer cells. After the development of a palpable tumor, tumor-bearing mice were orally treated with edelfosine (EDLF) (30 mg/kg, once-daily) or with an equal volume of water vehicle (Control). After completion of the in vivo assays (33 days, Capan-2; 48 days, HuP-T4), a drastic reduction in the tumors was observed following oral edelfosine treatment. Microscopic views of paraffin sections of tumors from control or edelfosine (EDLF)-treated Capan-2-bearing mice showed a remarkable staining of active caspase 3 (arrows), indicating the induction of apoptosis (**b**), and a dramatic upregulation of CHOP/GADD153 (arrows), indicating the induction of ER stress (**c**) in edelfosine-treated mice. Images taken from [132].

Further support for the involvement of the ER stress in the proapoptotic action of edelfosine in tumor cells derives from in vivo data of pancreatic cancer xenograft models. Oral administration of edelfosine (30 mg/kg of body weight) drastically reduced the size of pancreatic tumors generated by inoculation of Capan-2 or HuP-T4 human pancreatic tumor cells in CB17 severe combined immunodeficient (SCID) mice [132] (Figure 10). Tumors isolated from the xenograft mouse models with SCID mice, orally treated with edelfosine, showed a dramatic increase in apoptotic markers as well as in CHOP/GADD153 staining following immunohistochemical analyses [132] (Figure 10). These data, involving ER stress in the antitumor action of edelfosine, are also reproducible in other tumors, such as in Ewing’s sarcoma [135].

Interestingly, normal human primary fibroblasts, the non-tumorigenic and hTERT immortalized human pancreatic ductal HPNE cell line, and normal hepatocytes were not significantly affected by edelfosine [184,185,222].

## 13. ER Stress and Mitochondrial Connection in the Induction of Apoptosis in Pancreatic Cancer Cells by the Ether Lipid Edelfosine

The induction of pancreatic cancer cell apoptosis by edelfosine involves the caspase-8-mediated cleavage of the integral membrane protein of the ER (BAP31) into the 20-kDa fragment p20-BAP31 [132], which directs proapoptotic signals between the ER and the mitochondria [140,223] through a release of Ca^2+^ from the ER, concomitant with an uptake of Ca^2+^ into the mitochondria, leading to cytochrome *c* release and cell death [129,140]. Gajate et al. [132] found that edelfosine induced a caspase-mediated apoptotic response (involving activation of caspases 3, 7, 8, and 9) in pancreatic cancer cells, through the cleavage of BAP31 into p20-BAP31, alterations in the ER calcium level, and release of cytochrome *c* from the mitochondria to the cytosol. Ectopic expression of Bcl-X_L_, acting as a mitochondrial safeguard, prevented cytochrome *c* release and apoptosis without inhibiting ER-stored Ca^2+^ release [132,216]. Furthermore, *bax*^−/−^*bak*^−/−^ double-knockout cells failed to undergo edelfosine-induced apoptosis, whereas wild-type cells were sensitive to the drug [216]. Wild-type and *bax*^-/-^*bak*^-/-^ cells displayed similar patterns of phosphatidylcholine and protein synthesis inhibition, despite their differences in drug sensitivity, suggesting that edelfosine-induced apoptosis is dependent on Bax/Bak, but phosphatidylcholine and protein synthesis inhibition seems not to be critical [216]. These data indicate that the mitochondria are indispensable for edelfosine-induced, ER-stress-mediated cell death.

The ER and the mitochondria interact through dynamic platforms known as mitochondria-associated membranes (MAMs) [224,225,226], which show higher levels of cholesterol compared to the rest of the ER [227,228], and provide an excellent scaffold for crosstalk and the transfer of lipids between the ER and the mitochondria [226,229,230]. Because edelfosine accumulates in lipid rafts [150,170,180,181,184,187,188,193,201,228], shows a high affinity for cholesterol [193,228,231,232,233], and is also able to target the mitochondria [176,210,228]—likely through the involvement of lipid rafts [210,228]—it could be envisaged a network of central subcellular organelles involved in cell demise regulation that could be mediated by lipid rafts. In this context, edelfosine represents an excellent tool to unveil this raft-mediated membrane–ER–mitochondria network that could modulate signaling processes and cell fate.

## 14. Novel Approaches for the Potentiation of ER-Mediated Apoptosis in Pancreatic Cancer Cells Induced by Edelfosine

The above data clearly indicate that edelfosine induces ER stress and UPR signaling—the latter being known as a pro-survival response intended to diminish the accumulation of unfolded and altered proteins and restore normal ER function. Preincubation of BxPC-3 pancreatic cells with dithiothreitol (DTT)—a well-known ER stress and UPR inducer—did not lead to a statistically significant reduction in edelfosine-induced apoptosis [132], further supporting the notion that persistent edelfosine-induced ER stress overrides protective UPR mechanisms, and eventually switches the cytoprotective role of UPR to cell-death-promoting mechanisms [132]. In this regard, we suggest that the induction of sustained ER-stress-mediated apoptosis by edelfosine could be further potentiated by its combined use with additional ER stress inducers.

Human neutrophils contain constitutively high levels of arginase-1 [234], and the release of this enzyme leads to arginine deprivation in the surrounding medium. Arginine depletion inhibits T-cell activation [235] and induces autophagy as a cytoprotective response to ER stress in human T lymphocytes [114]; this could lead to the generation of an immune-privileged site for the tumor [236]. Nevertheless, cancer cells are more susceptible to arginine deprivation than their non-cancerous counterparts [237,238], and pancreatic cancer cells behave as one of the most sensitive cell types to the absence of arginine in the culture medium [239]. Recent data show that neutrophils are emerging as double-edged swords in cancer, being associated with both cancer progression and immunosurveillance against tumors, and can therefore either promote or inhibit cancer, and could play a major role in metastasis [236,240,241]. Based on RNA silencing experiments and biochemical approaches, we have recently found that the release of neutrophil-derived arginase-1 leads to arginine depletion which, in turn, induces ER stress in pancreatic cancer cells through the activation of the PERK→eiF2α→ATF4→CHOP axis, and eventually leads to cell death [239]. We have recently found that either arginine depletion or neutrophil-released arginase-1 highly potentiates the antitumor action of the ER-targeting antitumor ether lipid edelfosine against pancreatic cancer cells [239], opening up new avenues for cancer treatment.

On the other hand, as stated above, ER stress can lead to a potent increase in autophagy as a cytoprotective response and as a survival mechanism [114,242,243]. Autophagy is widely monitored through immunoblot analysis by the conversion of microtubule-associated protein 1 light chain 3 (LC3), which is located in the cytosol (LC3-I), to its phosphatidylethanolamine-conjugated form (LC3-II), which is membrane-bound and is located in the autophagosomal membrane, as the amount of LC3-II is correlated with the abundance of autophagosomes [244]. Edelfosine was found to induce a potent autophagic response, as assessed by an increase in the amount of LC3-II in BxPC-3 and MiaPaCa-2 human pancreatic cancer cells [222]. Pretreatment with the autophagy inhibitors chloroquine, bafilomycin A1, and 3-methyladenine potentiated the apoptotic cell death promoted by edelfosine, as assessed by flow cytometry analysis [222]. Thus, further induction of persistent ER stress along with blockade of autophagy, as a compensatory cytoprotective mechanism, could provide new therapeutic approaches to treat pancreatic cancer.

## 15. Outlook

**Figure 11:**
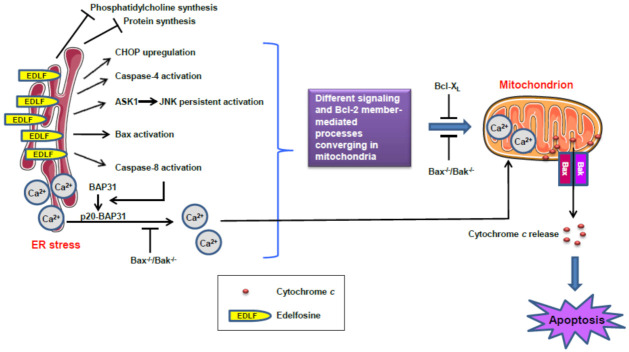
Schematic model of the ER’s involvement in the antitumor action of edelfosine in pancreatic cancer cells. Edelfosine is selectively taken up by tumor cells and accumulates in the ER. This targeting of the ER by edelfosine leads to a persistent ER stress response characterized by the different biochemical processes depicted in the schematic diagram. These events lead to apoptosis through a mitochondria-dependent process. See text for details.

Taking together the different studies carried out with the antitumor ether lipid edelfosine shown above, we can envisage a novel approach in the treatment of pancreatic cancer. Taking advantage of the readiness of pancreatic cancer cells to undergo ER stress due to their prominent and active ER, we might hypothesize that the use of an antitumor drug that shows a selective uptake for pancreatic cancer cells and accumulates in the ER of the pancreatic cancer cells could trigger a persistent ER stress response, leading eventually to tumor cell apoptosis. Importantly, the APL edelfosine meets these requirements—namely, it is selectively taken up by the cancer cells, sparing their non-cancerous counterparts, and interacts directly with the ER, accumulating in the ER and leading to persistent ER stress, which inevitably results in cell death. Figure 11 depicts a model for the involvement of ER in edelfosine-induced apoptosis, and highlights the importance of the accumulation of the APL in the ER, leading to sustained ER stress, which eventually leads to tumor cell apoptosis. The mitochondria are crucial for the above edelfosine-induced cell death initiated by ER stress. A number of different drugs (e.g., bortezomib, cisplatin, cannabinoids) can induce an ER stress response in an indirect way, affecting ER-mediated processes, but edelfosine physically accumulates in the ER, leading to a very potent and persistent ER stress response and, eventually, to apoptosis.

This targeting of the ER by edelfosine represents a novel approach in the treatment of the thus-far undruggable pancreatic cancer. As stated above, pancreatic cancer is usually diagnosed at an advanced stage, showing metastatic spread at diagnosis, and a dismal prognosis. The particular features of pancreatic cancer, herein discussed, and the inefficient current chemotherapy that only improves life survival by an average of a few months, show the urgency of the need to develop more effective treatments.

Interestingly, because the induction of ER stress generates autophagy as a compensatory cytoprotective response to keep tumor cells alive, the combination of ER stress inducers plus autophagy inhibitors could represent a promising strategy in the treatment of this thus-far incurable cancer.

Preclinical studies with xenograft animal models rendered excellent results in promoting cell death in pancreatic cancer cells via an ER-mediated process following oral edelfosine treatment. The selectivity of edelfosine for cancer cells means that this antitumor ether lipid accumulates in the tumor in in vivo assays [180,181], and lacks significant toxicity [183]. 

We have very recently found that the release of arginase-1 from neutrophils could promote an ER-stress-mediated cell death process in pancreatic cancer cells [239]. Because of their ability to interact with cancer cells, as well as their outstanding capability to travel to different tissues [236], neutrophils could be used as vehicles to carry arginase-1 to the metastatic site. Combination with neutrophil-released arginase-1 clearly potentiates the antitumor activity of edelfosine against pancreatic cancer cells [239]. Because there is a significant neutrophil infiltration in pancreatic cancer [245,246,247,248,249,250,251,252], this could open a new approach to analyze in order to potentiate the antitumor activity of the ER-targeting drug edelfosine. Taking all of the above evidence into consideration, we postulate that further induction of persistent ER stress by combination therapy, as well as blockade of autophagy, acting as a compensatory cytoprotective mechanism, could provide new therapeutic approaches to treat pancreatic cancer. These data highlight how the direct targeting of the ER, leading to a physical antitumor drug accumulation in the ER with a concomitant and persistent ER stress, could be a promising therapeutic approach in the therapy of pancreatic cancer, provided these new drugs are selective for tumor cells, so that non-cancer cells are not affected.

An increasing number of newly synthesized drugs—including topsentin, thiadiazole, and indole derivatives, which inhibit protein kinases and modulate key regulators of epithelial-to-mesenchymal transition—are being tested against pancreatic cancer cells [253,254,255,256]. On the other hand, amphiphilic pyrrolidine derivatives also show antitumor activity against a number of human pancreatic cancer cell lines, which is potentiated by the inhibition of autophagy [222]. The diversity in chemical structures suggests that different molecules can be targeted, but no effective treatment for pancreatic cancer has been developed thus far. The antitumor ether lipid edelfosine targets cellular membranes, is preferentially taken up by tumor cells, and accumulates in the ER, triggering sustained ER stress and subsequent apoptosis. These particular features make edelfosine a promising drug for the treatment of pancreatic cancer, and structure–activity relationship studies could provide new insights into its mechanism of action and unveil the chemical structure required for selective tumor cell uptake and ER targeting, which might contribute to the generation of improved drug derivatives against pancreatic cancer.

## 16. Conclusions

The existing chemotherapeutic armory against pancreatic cancer is ineffective and, hence, the pancreatic cancer survival rates have remained relatively unchanged over the past 50 years. The antitumor ether lipid edelfosine—the prototype molecule of a family of synthetic drugs collectively known as alkylphospholipid analogs—is a 2-lysophospholipid analog that shows selective proapoptotic activity against a wide variety of human tumors, including pancreatic cancer, because of its preferential uptake by cancer cells, whereas non-cancerous cells are spared. A hallmark of pancreatic cells is their abundant and active ER to cope with their high rates of protein synthesis. Thus, the ER can be an interesting target for pancreatic cancer therapy. Edelfosine is taken up by pancreatic cancer cells and accumulates in the ER, leading to persistent ER stress and subsequent apoptosis. Edelfosine is orally administered, lacks significant toxicity, and can be considered to be a paradigmatic drug for an ER-targeted therapy. Edelfosine shows a potent in vitro and in vivo antitumor activity against pancreatic cancer through an ER-mediated process. This opens a new paradigm in pancreatic cancer chemotherapy that involves the selective uptake of an ER-targeted drug by the tumor cells, taking advantage of the extremely high protein synthetic activity of pancreatic cells. It is tempting to envisage that this new approach could avoid the high toxicity, and improve the poor results, shown by the current treatment of pancreatic cancer.

## Abbreviations

AELs	Antitumor ether lipids
ALPs	Alkyl-lysophospholipid analogs
APLs	Alkylphospholipid analogs
ATLs	Antitumor lipids
ATF6	Activating transcription factor 6
BAP31	B-cell receptor–associated protein 31
BiP	Binding immunoglobulin protein
BODIPY	4,4-Difluoro-4-bora-3a,4a-diaza-*s*-indacene; boron-dipyrromethene
CHOP	CCAAT/enhancer-binding protein homologous protein
ER	Endoplasmic reticulum
ERK	Extracellular signal-regulated kinase
GADD153	Growth-arrest and DNA-damage-inducible gene 153
IRE1	Inositol-requiring enzyme 1 (IRE1)
FOLFIRINOX	Folinic acid, 5-fluorouracil, irinotecan, and oxaliplatin
GRP78	78-kDa glucose-regulated protein
HDI	Human Development Index
JNK	c-Jun NH_2_-terminal kinase
MAMs	Mitochondria-associated membranes
MAPK	Mitogen-activated protein kinase
MEK	MAPK/ERK kinase
MIR	Mortality-to-incidence ratio
MM	Multiple myeloma
mTOR	Mammalian target of rapamycin
Nab-paclitaxel	Nanoparticle albumin-bound paclitaxel (Abrazane)
PanNET	Pancreatic neuroendocrine tumor
PDAC	Pancreatic ductal adenocarcinoma
PERK	Protein kinase RNA (PKR)-like ER kinase
PI3K	Phosphoinositide-3-kinase
RAF	Rapidly accelerated fibrosarcoma
RFP	Red fluorescent protein
SCID	Severe combined immunodeficiency
UPR	Unfolded protein response
